# Study of Stem Cells in Human Milk

**DOI:** 10.7759/cureus.23701

**Published:** 2022-03-31

**Authors:** Shailaja Mane, Satvika Taneja, Jyothsna Sree Madala, Sharad Agarkhedkar, Meghna Khetan

**Affiliations:** 1 Pediatric Medicine, Dr Dnyandeo Yashwantrao Patil Medical College, Hospital and Research Center, Dnyandeo Yashwantrao Patil Vidyapeeth (DPU), Pune, IND

**Keywords:** research, multilineage differentiation, mesenchymal stem cells (mscs), multipotent nature, breast milk

## Abstract

Stem cells are cells that have the ability to self-renew into an undifferentiated cell state, which can further delineate into distinct cell types. There are various sources of stem cells in the human body; some of them include cord blood, placental tissue, bone marrow, adipose tissue, dental pulp, etc.

Breast milk could become an important source of stem cells in the near future because of its non-invasive isolation technique. Based on this nature, this study was conducted to isolate stem cells from breast milk and to show further potential implications of these cells. The total number of cells isolated from the milk ranged from 1.5 × 10^5 ^cells to 3 × 10^5 ^cells. As there was prolongation in the lactation period, the number of cells in the milk lowered significantly. There was no significant difference in the cell count in various gestational age groups. The cytochemistry analysis of these cells with their specific cell markers confirmed the presence of a homogenous population of mesenchymal stem cells. Further differentiation of these breast milk stem cell analyses showed transformation into adipocytes, chondrocytes, and osteoblasts in different culture mediums. So the presence of mesenchymal stem cells in human milk, which are multipotent in nature, makes it an important source of stem cells for further regenerative therapies, tissue culture techniques, and gene therapies. Due to this nature, these cells can be redirected to produce various tissues in the human body.

## Introduction

Human milk is designed for the nutritional and immunological needs of infants. It contains a diverse microbiome in addition to macro and micronutrients. It contains immunoglobulins, lactoferrin, lysozymes, cytokines, and other immunological components that provide active and passive immunity to the newborn. In humans, the normal reproduction process takes a long time. During embryonic life, the mother provides immunological factors to the developing foetus, which protects the foetus from infection and aids in the formation of its intestinal mucosa and gut flora. In normal circumstances, leucocytes account for 14-71% of total cells isolated from breast milk; however, during an infection, this percentage can rise to 94% [1].

Along with the presence of immunological, nutritional, and metabolic factors in human milk, unique and diversified cells are present, called stem cells. Breast milk stem cells generally exist in lower numbers in a healthy mammary gland when in a resting state, but during pregnancy and breastfeeding, they are produced in increased numbers. In mammary tissue, they can differentiate into alveolar, ductal, and myoepithelial cells. The stem cells discovered in breast milk are embryonic in nature and have markers like nestin, cytokeratin, OCT 4, SOX 2, NANOG, SSEA 4, and TRA-1. Mesenchymal in nature, represented by markers CD44/90/271/146/105, as well as hematopoietic in nature, with CD34 as a marker [2].

The isolation of stem cells from human milk is demonstrated in this study. In addition to the isolation, a comparison of stem cell count on consecutive postnatal day milk samples from term and preterm mothers was demonstrated. We also demonstrated the culture characteristics and ability of these isolated cells to differentiate further in terms of cell surface markers.

## Materials and methods

Collection of samples

Thirty mothers delivered in our hospital were chosen, ten of whom were delivered at preterm gestation and twenty of whom were at term, with normal postnatal maternal and neonatal clinical health and whose babies were started on breast milk after birth. The mothers were counseled about the study in a language they could understand. The study was carried out after the mothers provided written informed consent. Dr. D. Y. Patil Medical College Hospital and Research Center-Institutional Ethics Sub-Committee issued approval IESC/PGS/2019/32.

The mothers were instructed to express 5 mL of breast milk using a rhythmical rolling motion of the thumb and index finger after washing the breast. The milk samples were collected on both postnatal days 3 and 5. In the early hours of the morning, milk samples were collected from the mothers. Within 15 to 20 minutes, the milk was collected in sterile containers and transported to the lab. Breast milk was collected and transported with care; sample collection was carried out under aseptic conditions.

Isolation, characterization, and differentiation of cells

Individual milk samples were diluted in DMEM (Dulbecco's modified Eagles medium), which also contains specific antibiotics to prevent contamination of the solution, and for separation, the solution was centrifuged at 1800 rpm for five minutes. Following centrifugation, the supernatant and pellet were separated. After discarding the supernatant, the remaining solution was centrifuged for five minutes at 1800 rpm after washing with saline phosphate buffer solution. The obtained supernatant was discarded once again, and the cell pellet was implanted in a tissue culture treatment plate containing DMEM medium. This culture medium also contained 10% heat-inactivated human umbilical cord blood serum. For better cell growth, the medium was changed every other day. Cells were passed using trypsin-ethylenediamine tetraacetic acid (EDTA) after they had reached maximum confluence. This method was used to isolate cells from breast milk.

The cells obtained in the previous passages were used in the subsequent studies for characterization. Cells grown on coverslips were fixed with 4% paraformaldehyde (PFA) for five minutes before being permeabilized with 50% methanol. The cells were then incubated in PBS for one hour with 5% bovine serum albumin (BSA), which aids in the removal of non-specific binding sites that interfere with the results. Antifade (Vecta shield) and 4′,6-diamidoino-2-phenylindole were used to mount the coverslips (DAPI). A confocal laser scanning microscope was used to examine these slides. DAPI was used to visualize the nuclei (Invitrogen). After being dislodged with 0.05% trypsin and 0.02% EDTA in PBS, cells from the final passages were removed and resuspended in DMEM. Cells were fixed in cooled 70% ethanol and incubated for one hour on ice with mouse anti-human FITC/phycoerythrin (PE) conjugated antibodies against CD34, CD45, CD73, CD90, and CD105, HLA DR (1:100 dilution). The cells were collected using a laser 488 nm flow cytometer, and the data were analyzed using BD Cell Search Pro software (BD Biosciences, Mississauga, ON, Canada). After the characterization of these cells, further studies were performed to know the differentiation potential of these obtained cells.

Differentiation studies

The stem cell clones obtained in the culture were passed through adipogenic induction and maintained in a medium during the initial passages (PT-3102A, Cambrex, East Rutherford, NJ). Later, these cells were mounted on a culture medium for chondrogenic and osteogenic differentiation and cultured at 37 °C for 24 hours to achieve optimal growth. These lineages were induced by substituting their respective differentiation kits for the growth medium (DMEM; PT-3003, PT-3002, and CC-3229). To confirm adipogenesis, oil red O stain was used; chondrogenesis was confirmed by Safranin-O stain; Alizarin Red S staining was used to confirm osteogenesis. The visualization of cell growth patterns was carried out following the application of appropriate growth media and staining techniques.

## Results

In this study, the mean (SD) age of the mothers who were included was 27.4 (3.5) years, with a minimum age of 20 and a maximum age of 35 years. The mean (SD) age of the mothers delivered at preterm gestation (<36.6 weeks) was 27.1 (3.4) years, and the mean (SD) age of the mothers delivered at term gestation (≥37 weeks) was 27.6 (3.6) years.

In the obtained results, we have demonstrated the difference in the cell count as the lactation goes on, that is, the difference in the cell counts in the milk samples on day 3 and day 5 samples. Table 1 shows that the mean (SD) cell count obtained from the samples collected on day 3 was 3.18 (0.14) and the mean (SD) cell count on day 5 was 1.28 (0.12).

**Table 1:** Mean cell count data Paired t-test, p-value - highly significant

Day of milk collection	Number of participants	Mean	SD	Mean difference (95% CI)	P-value
Day 3	30	3.18	0.14	1.89 (1.83–196)	<0.001
Day 5	30	1.28	0.12		

**Figure 1:**
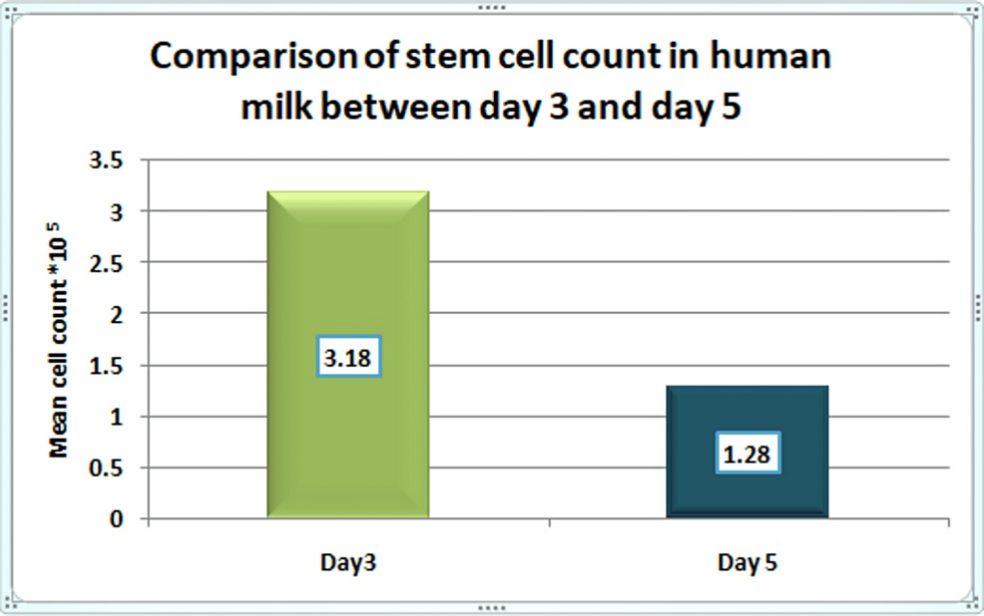
Mean cell count

Figure 1 shows that the mean (SD) cell count obtained from the 10 preterm mothers on the postnatal day 3 sample was 3.17 (0.13), while the remaining 20 term gestation mothers had a mean (SD) cell count on day 3 of 3.18 (0.15). The mean (SD) cell count obtained from the 10 preterm mothers on the postnatal day 5 sample was 1.33 (0.1). The remaining 20 term gestation mothers had a mean (SD) cell count on day 5 of 1.28 (0.13).

**Figure 2:**
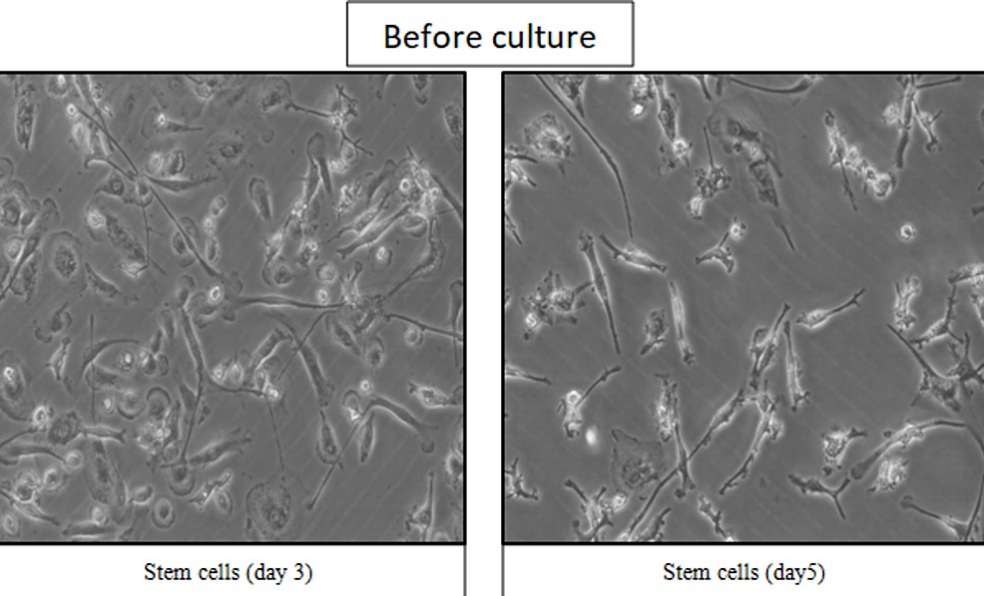
Stem cell counts of day 3 and day 5 breast milk samples before cell culture

Further culture and differentiation data reciprocate the summary of the cell data of total samples obtained from mothers on both post-natal days. The cells obtained from the post-natal milk samples before the culture (Figure 2) showed the morphological appearance of an epithelial cell-like picture.

**Figure 3:**
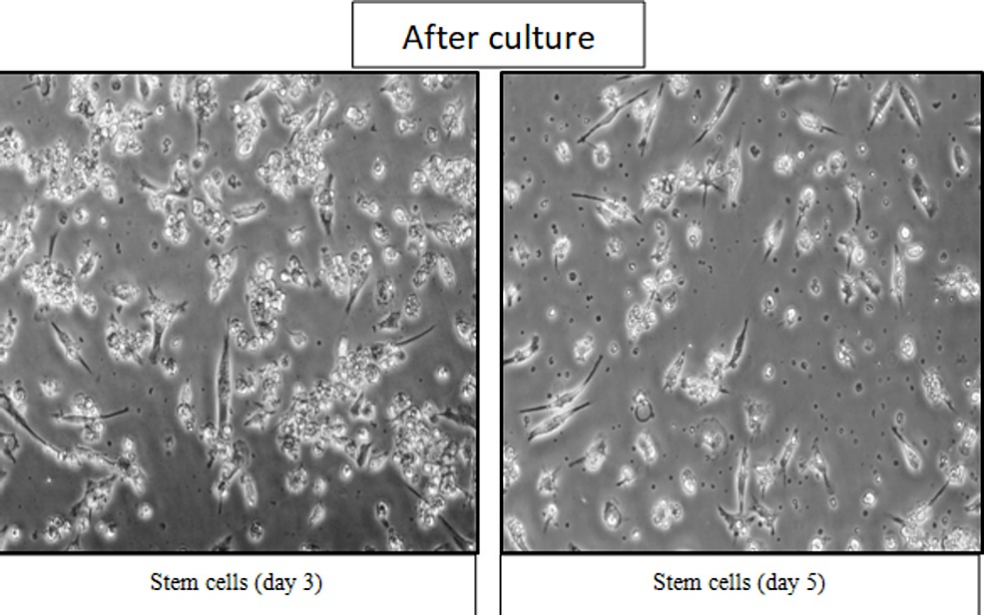
Stem cell counts of day 3 and day 5 breast milk samples after cell culture

These cells, which underwent culture in the second week, displayed a fibroblast-like appearance (Figure 3). This transformation is mainly because of the development of the mesenchymal stem cell population. The maximum confluence was reached within a week. The morphology of cells transformed from epithelial to fibroblast-like after culture in the second week.

**Figure 4:**
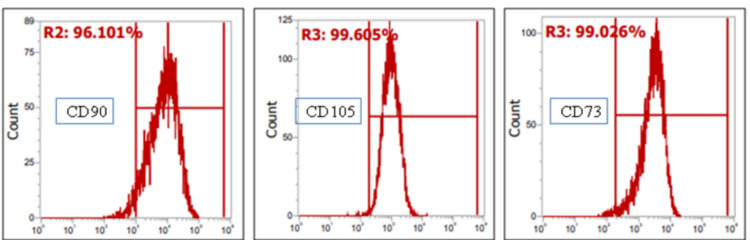
Analysis of breast milk stem cells with various mesenchymal cell surface markers

A study of immunofluorescence and cytochemistry for specific cell surface markers revealed that isolated human breast milk cells from early passages expressed mesenchymal cell surface markers (Figure 4). The cells expressed cell-specific surface markers CD 73 (99.026%), CD 90 (96.101%), and CD 105 (99.605%). These cells did not express CD34, CD45, and HLA-DR, confirming their identity as MSCs. The cells isolated from the fresh milk contained a very minimal percentage of positive cell surface markers, while they were significantly increased by up to 99% by the final passages. These results indicate the presence of an abundant number of mesenchymal stem cells in human milk.

**Figure 5:**
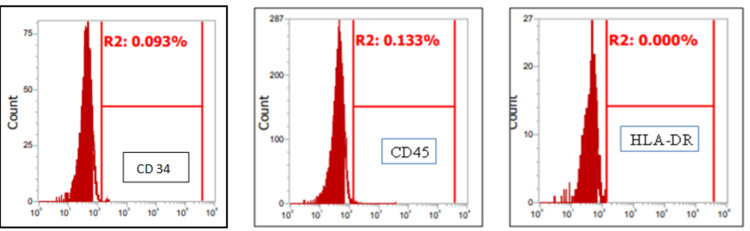
Analysis of breast milk stem cells with various mesenchymal cell surface markers

The cells obtained from the final passage showed negative expression of surface markers like CD 45 (0.133%), CD 34 (0.093%), and human leukocyte antigen DR isotype (0.000%) (Figure 5).

This shows the presence of mesenchymal stem cells in breast milk, which significantly increases in percentage after culture. Differentiation studies were performed with the help of different media, which were differentiated into adipogenic, chondrogenic, and osteogenic lineages. This shows the potential nature of stem cells to differentiate into various cell lineages. After incubation of breast milk stem cells for almost three to four weeks, a change in the morphology and characteristics of the cells was seen.

**Figure 6:**
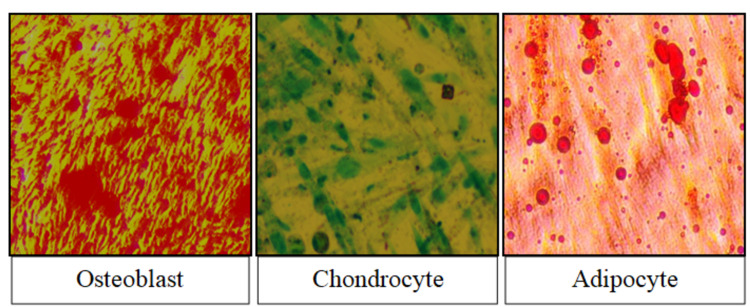
Analysis of differentiation of breast milk mesenchymal stem cells

The osteogenic differentiation of cells was confirmed by the transformation of cells into a cuboidal shape with mineralization that was stained positively by Alizarin red S, indicating direct evidence of calcium deposits. Chondrogenic differentiation is visible in the cells as the transformation of spindle-shaped cells into large round cell aggregates and the accumulation of proteoglycans found in cartilage, which were stained positive with Safranin 0. The cells' morphology changed from spindle to round to oval, with the appearance of numerous large round intracytoplasmic lipid droplets stained positive by oil red O, confirming the adipocyte phenotype (Figure 6).

This confirms that breast milk stem cells can be differentiated into adipogenic, chondrogenic, and osteogenic cell lineages, which confirms the differentiation capability of mesenchymal stem cells of human milk.

## Discussion

According to this study, the cell count decreased from postnatal day 3 to postnatal day 5 in milk samples. This demonstrated that the cell count decreased significantly as lactation progressed. Similar studies conducted by different researchers, Li et al. [3], Tang et al. [4], and Indumathi et al. [5], showed similar results to our study that maximum milk cell population occurs in the early days of lactation. Trend et al. [6] hypothesized in their study that this decrease would be due to an increase in the quantity of breast milk as lactation progressed, and thus milk dilution occurs with a relative decrease in the cells per unit of measured volume. There was no significant difference in cell counts between gestational age groups in postnatal day 3 and postnatal day 5 milk samples. 

In a study to look at the different characteristics of milk, Li et al. [3] discovered that there was no difference in the percentages of immune cells, mesenchymal stem cells, and SOX2 cells at different stages of lactation and gestational age groups (28 weeks, 28.1-32 weeks, 32.1-37 weeks, and 37.1-41 weeks). Similar findings from Kaingade et al. [7] revealed that different gestational age groups had no effect on cell counts. Another study conducted by Keller et al. [8] hypothesized that gestational age might influence cell counts but did not clearly demonstrate the reason for the difference. This study demonstrated that proper culture techniques can significantly increase cell count with a shorter time duration and very simple, non-invasive techniques. Several other studies were conducted to demonstrate the presence of stem cells in breast milk and their ability to divide into mesenchymal stem cells.

Patki et al. [9] demonstrated that mother's milk contains a high concentration of multipotent stem cells. Hosseini et al. [10] demonstrated that breast milk stem cells have pluripotency and can develop into different neural lineages. The different lineages he demonstrated were neurons, oligodendrocytes, and astrocytes. According to Pichiri et al. [11], the markers for mesenchymal stem cells were CD 44, followed by CD 90, and CD 105. Normal stem cells are present in breast milk, but after appropriate culture techniques in adequate medium, the number and potentiality of stem cells significantly increase, as confirmed in our study and similar findings demonstrated by Indumathi et al. [5]. In comparison to fresh milk samples, the differentiation potential of these cells was also significantly increased.

When it comes to the history of stem cell isolation, it was first elaborately described by Cregan et al. [12] in 2007 when he discovered the stem cell marker Nestin in the term breast milk, but he could not explain the important feature of the differentiation potential of these cells, which was later confirmed by Fan et al. [13]. Thomas et al. [14,15] identified the cell markers CD49f and p63, which are progenitor markers from breast milk-derived cells. Later studies were conducted to demonstrate various characteristics of these stem cells in relation to maternal and neonatal characteristics, as well as prenatal and delivery complications. Many more studies are being conducted to use these stem cells in various disorders, therapies, and regenerative therapies [9-11].

This stem cell therapy is now thought to be a therapeutic option and the only treatment for many long-term disorders. Stem cells are found in many other organs of the body, but it is very simple and non-invasive to collect stem cells from breast milk. The main advantage of this technique is that a large number of cells can be obtained from a small amount of milk, and further appropriate culture increases the characteristics of these cells within a limited time frame.

Implications

The research community wishes and hopes to incorporate these stem cells into the treatment of various organ and system disorders in the body. Several studies have revealed that these stem cells can be used to treat blood disorders, liver disorders, and neuronal disorders. The stem cells derived from the mother's milk can be cultured and stored for future use in her own baby for any health disorders or autoimmune conditions. This is known as autologous stem cell therapy, and it is the most important thing we can accomplish in the near future to control various disorders and develop various therapeutic approaches. [16]

Many studies have shown that these stem cells can be differentiated into a variety of lineages, including mammary cells (both myoepithelial and luminal cells), cardiomyocytes, pancreatic beta cells, neuronal precursor cells (which include cells required for normal myelination, metabolic functioning, and defense mechanisms), osteocytes, adipocytes, chondrocytes, and hepatocytes. As a result, proper culture techniques and the production of these stem cells have significant future implications in the fields of stem cell transplantation techniques, gene therapy, and tissue culture techniques [17].

## Conclusions

These human milk-derived mesenchymal stem cells have the potential to be "reprogrammed" to generate a wide range of human tissues, making them useful in a variety of therapies. In comparison to fresh milk samples, there will be a significant increase in the expression of stem cells after culturing in the appropriate culture medium. The added benefit of these cells is that their isolation remains a simpler and less invasive technique, whereas many other organs produce stem cells but the extraction technique is difficult and invasive. This study emphasizes the benefits of stem cells, and because breast milk contains the most stem cells in the early days of lactation, early initiation and promotion of exclusive breastfeeding are recommended for all newborns in order to reduce neonatal morbidity and mortality.
